# Abdominal aortic aneurysm and its association with duodenal obstruction: aortoduodenal syndrome

**DOI:** 10.1259/bjrcr.20200040

**Published:** 2020-06-30

**Authors:** Kalind Parashar, Darshan Gandhi, Pankaj Nepal, Joshua Sapire, Kriti Ahuja, Imran Siddiqui

**Affiliations:** 1 Department of Radiology, St. Vincent's Medical Center at Hartford Healthcare 2800 Main Street, Bridgeport, CT 06606, USA; 2 Department of Radiology, Northwestern Memorial Hospital, Northwestern University Feinberg School of Medicine, 676 N St. Clair St, Suite 800, Chicago, IL 60611, United States; 3 Maulana Azad Medical College, New Delhi, Delhi 110002, India; 4 Department of General Surgery and Surgical oncology, St. Vincent's Medical Center at Hartford Healthcare 2800 Main Street, Bridgeport, CT 06606, USA

## Abstract

Proximal small bowel obstruction in the region of the duodenum is an uncommon clinical entity. Our case, which involves obstruction of the third portion of the duodenum due to an abdominal aortic aneurysm (AAA), is even more unusual. A review of the relevant literature regarding duodenal obstruction due to extrinsic compression includes features that differentiate aortoduodenal syndrome from superior mesenteric artery syndrome. Management of these conditions range from conservative to surgical, of which now includes a more recent role of metallic stents in some instances.

## Introduction

Aortoduodenal syndrome, defined as obstruction of the third portion of the duodenum by a large abdominal aortic aneurysm (AAA), is a very rare cause of intestinal obstruction. This condition was first described by William Osler in 1905 with approximately 30–40 cases reported within the literature since that initial description.^1,2^ The mechanism of obstruction involves direct compression of the duodenum by the aneurysm itself. Patients typically present with abdominal pain, nausea and vomiting. Additional symptoms, that is, weight loss and malnutrition, have also been reported. Treatment options to relieve the obstruction include non-surgical management like correction of nutritional status or surgical management. We present a case report of a patient successfully diagnosed and treated for this rare pathology due to an AAA.

## Case report

**Figure 1.**
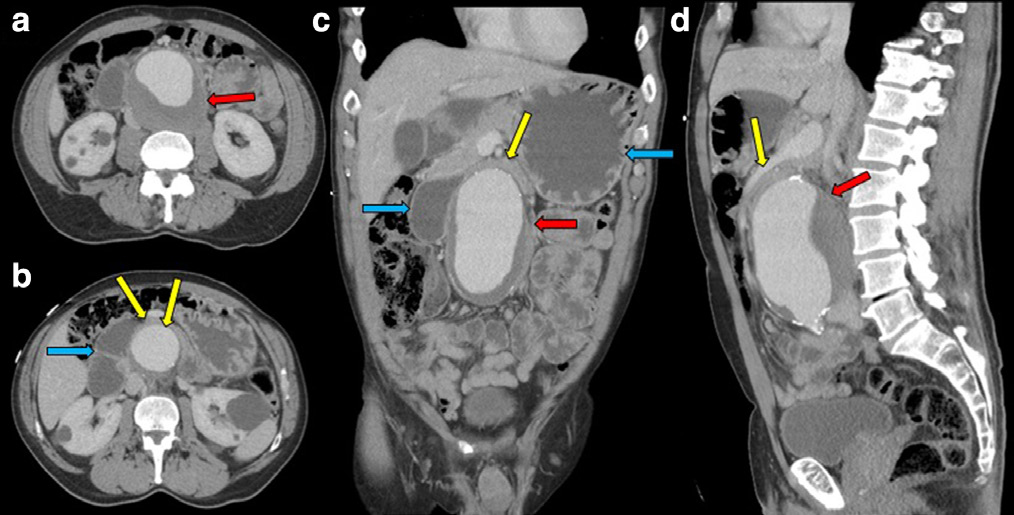
CT abdomen and pelvis postcontrast venous phase with axial views in figure 1A and 1B, coronal view in figure 1C and sagittal view in figure 1D, showed partially thrombosed large abdominal aortic aneurysm (Fig 1A,1C,1D) (red arrows) compressing third portion of duodenum between aneurysmal aorta and superior mesenteric artery (Fig 1B,1C,1D) (yellow arrows) and fluid-filled dilatation of proximal duodenum and stomach (Fig 1B and 1C) (blue arrows).

64-year-old male with a previous medical history of prior tobacco use, gastroesophageal reflux disease and hypertension presented to the emergency department with recurrent and worsening epigastric abdominal pain for 1 month. The pain was associated with episodic bilious vomiting and which also contained food particles. The patient’s history was negative for recent unintended weight loss, hematemesis, melena or known malignancies. Laboratory findings revealed an elevated lipase level of 1300 U/L and leukocytosis of 18.0×10^9^/L. Based on these initial parameters, the clinical suspicion was acute pancreatitis, although there was no family history of pancreatitis and no personal history of alcohol abuse. The patient subsequently underwent a contrast-enhanced CT scan of the abdomen and pelvis using a dedicated pancreas protocol which included soft tissue and bone algorithms as well as reformatted/MPR images. The total amount of intravenous contrast administered was 95 ml omnipaque 350. Oral contrast was not administered. The CT demonstrated marked dilatation of both the stomach and duodenum with an abrupt transition point corresponding to the location of the third portion of duodenum secondary to mass effect by a large AAA (Figure 1). Additional findings included diffuse atherosclerotic calcifications of the aorta with aneurysmal dilation measuring 9 cm in transverse and 11 cm craniocaudal dimensions. There was no CT evidence of extravasation of contrast or other signs of impending aneurysmal rupture. The ER clinician consulted both vascular and gastrointestinal surgery. Due to the elevated lipase levels and the acute bowel obstruction, vascular surgery was not a viable management option at the time of presentation. The benefits and risks of open aortic surgery versus endovascular repair (EVAR) were discussed in detail and, following nasogastric tube decompression, the patient underwent EVAR of the AAA as the preferred treatment option.

**Figure 2.**
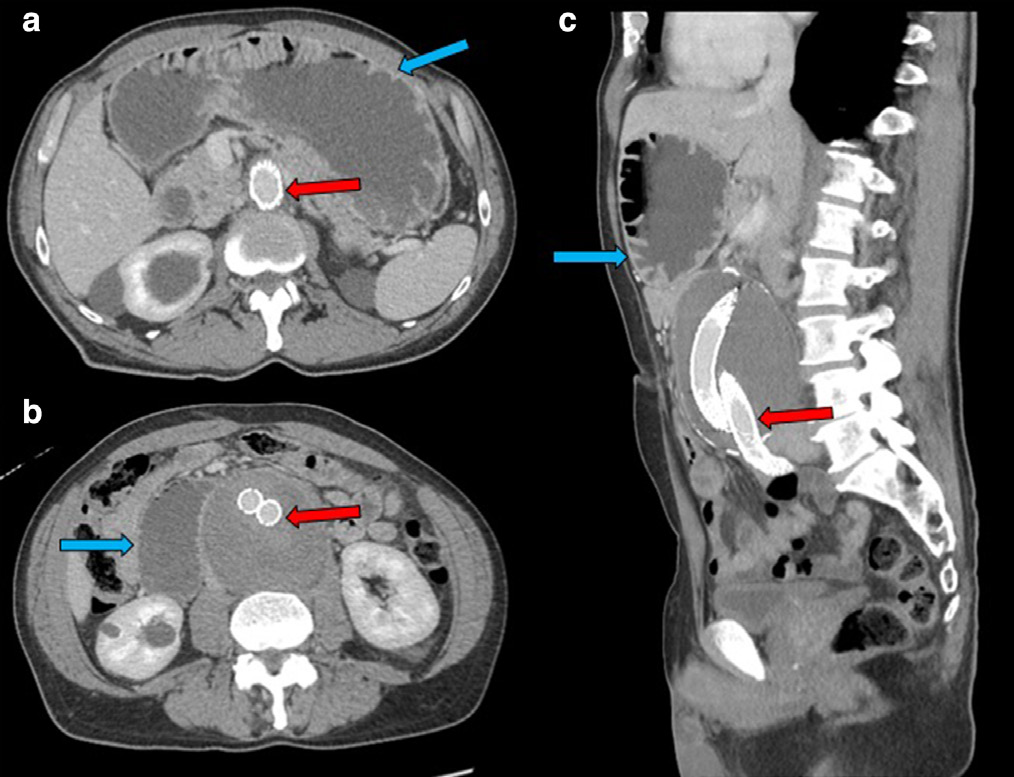
CT abdomen and pelvis postcontrast venous phase with axial views in figure 2A and 2B, and sagittal view in figure 2C, showed status postabdominal aortic aneurysm endovascular stent graft repair (Fig 1A, 1B, 1C) (red arrows) with persistent fluid filled dilatation of proximal duodenum and stomach (Fig 1A, 1B, 1C) (blue arrows).

**Figure 3.**
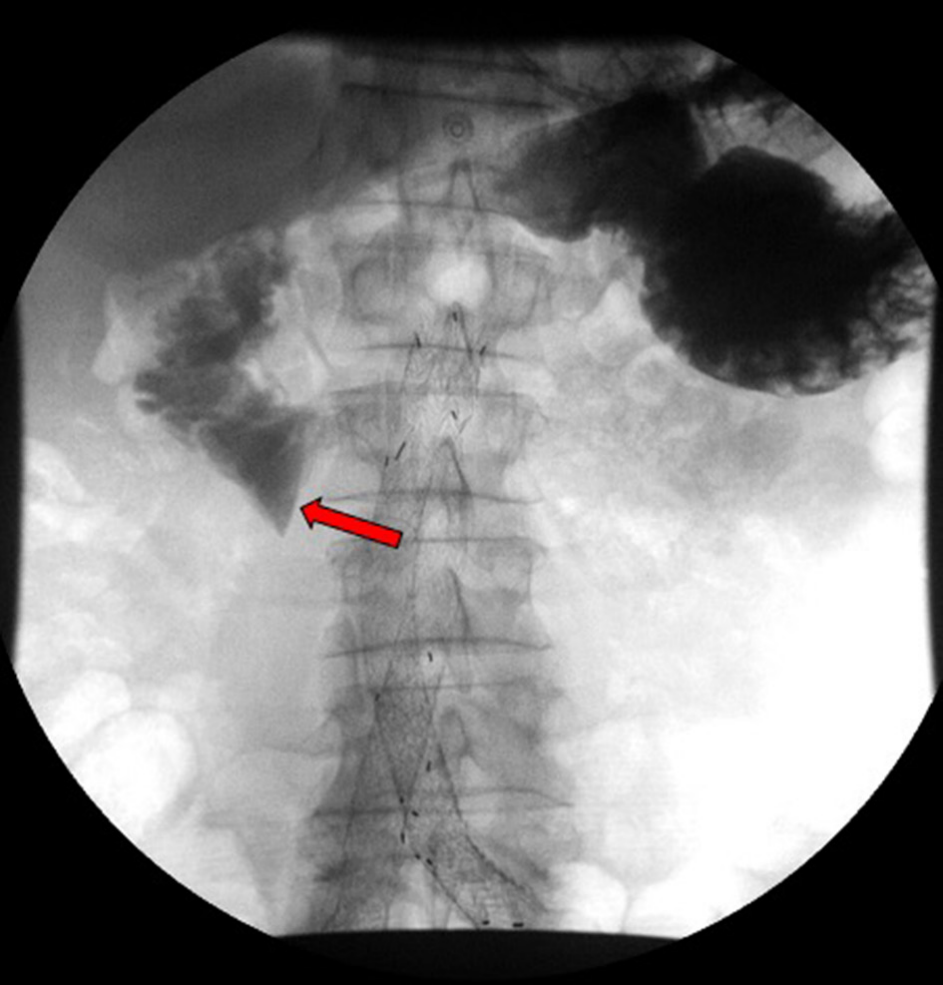
Fluoroscopic small bowel evaluation with gastrografin contrast, status postendovascular stent repair, revealed persistent gastrografin contrast in proximal third portion of the duodenum (red arrow) with no contrast traversing in distal small bowel loops in frontal view.

**Figure 4.**
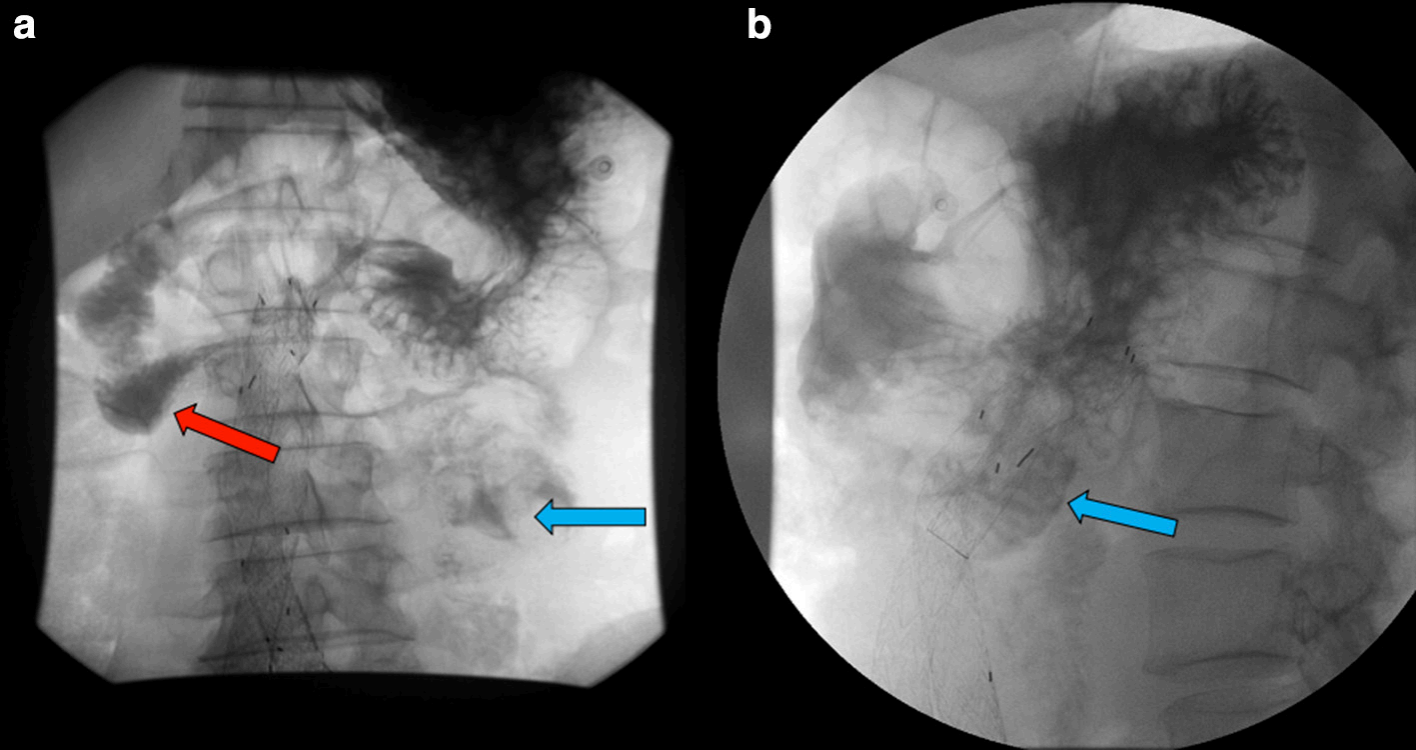
Fluoroscopic small bowel evaluation with gastrografin contrast, status postrobotic Roux-en-Y gastrojejunostomy, revealed delayed yet manifestation of transition of gastrografin contrast in the jejunal loops in left mid abdomen (blue arrows) (Fig 4A frontal view and 4B lateral view).

The patient was discharged home after successful EVAR. Despite this, however, symptoms of epigastric pain and vomiting were not relieved. Within the month, the patient presented two more times to the emergency department with clinical features of persistent duodenal obstruction and similar imaging features on repeat CT scans including stable size of the AAA (Figures 2 and 3). Due to symptoms of recurrent obstruction, and a determination that the patient could be a candidate for bypass which would allow gastric emptying in the form of a gastrojejunostomy or a duodenojejunostomy, he subsequently underwent successful robotic Roux-en-Y gastrojejunostomy which bypassed the obstruction. Following the bypass procedure, the patient underwent fluoroscopic evaluation with water-soluble oral contrast material (Omnipaque 240). The procedure was performed with the patient in upright, supine and oblique positions. These images demonstrated contrast within the jejunal loops within the mid-abdomen on the left and a persistent delay in transition, but which was improved when compared with pre-Roux-en-Y gastrojejunostomy (Figure 4). The patient tolerated a gradual advance in diet and was discharged home. Importantly, the patient’s symptoms were relieved at the follow-up clinic visit 4 months after surgery. The patient continues to have regular follow-up with the vascular surgeon.

## Discussion

While rare, this type of pathology does have a male predominance with most patients being over the age of 60 years and is unusual before the age of 40. One should raise suspicion for aortoduodenal syndrome when there is a combination of a pulsatile abdominal mass or known AAA with symptoms of gastric outlet obstruction.

The AAA directly compressing the duodenum either against the superior mesenteric artery (SMA) or the abdominal wall is most probable mechanism of obstruction.^3^ The defined pathogenesis of the aortoduodenal syndrome remains unclear^4^ although most of the cases reported in the literature were due to aneurysms larger than 7.8 cm and it is postulated to occur as a result of two anatomical factors: the first being the fixed nature of the mid-to-distal duodenum, which is retroperitoneal, and the second being the location of the SMA.^5^

To establish the diagnosis of aortoduodenal syndrome and rule out other causes of gastric outlet obstruction, for example a mass, CT with intravenous contrast should be done. This can be followed by either upper gastrointestinal (GI) endoscopy or upper GI contrast-enhanced imaging.^6^ CT angiography (CTA) of the abdominal aorta and mesenteric arteries is invaluable in preoperative diagnosis, surgical road-map planning and the relationship of the duodenal obstruction with respect to the adjacent mesenteric arteries. CT can also evaluate complications of the AAA such as leak, rupture and fistulous communication with the adjacent bowel which can affect endoscopic versus open surgical management. Given the scarcity of this entity, imaging findings of aortoduodenal syndrome are not established, and few cases have been reported with abrupt transition of the dilated stomach and proximal duodenum at the third portion of the duodenum with associated edema of the duodenal wall.^7^ Both surgeons and radiologists should be vigilant in cases of AAA associated with duodenal obstruction on routine CT abdomen with contrast studies for evaluation of bowel obstruction. In our experience, a fluoroscopic upper GI study may not be helpful emergently but is useful for follow-up after surgery or in cases managed conservatively.

Interesting correlations and differences as causes of extrinsic duodenal compression are found between aortoduodenal syndrome and SMA syndrome, also known as Wilkie syndrome.^8^ Specifically, with respect to the demographics of aortoduodenal syndrome, the patient with SMA syndrome is usually a young female with low body weight and a paucity of intraperitoneal fat. CT findings with SMA syndrome include an aortomesenteric angle of <38 degree or aortoduodenal distance of <10 mm.^8^ Interestingly, our patient had an aortoduodenal distance of <10 mm, but the duodenal obstruction was already being caused by the large aneurysmal aortic component, thus diagnosed as aortoduodenal syndrome. Pal et al demonstrated a case report in the literature where SMA syndrome was associated with AAA. That patient, however, was diagnosed with SMA syndrome because the duodenal obstruction was due to a narrow aortomesenteric angle, diagnosed radiographically and intraoperatively, and the patient also had a low BMI as well as a paucity of intraperitoneal fat.^9^

Management for this condition range from conservative treatment to surgical decompression, typically of the associated adhesions, seroma, or hematoma. Surgery includes a pre-operative assessment with endoscopy to exclude other common causes of obstruction. To lessen the risk of pulmonary aspiration, nasogastric decompression is essential. Primarily, treatment was palliative gastric bypass before the emergence of aortic surgery. Now most patients with aortoduodenal syndrome are managed by aortic graft placement.^7,10^ Morbidity due to aortoduodenal syndrome is usually due to aspiration pneumonia, renal failure, significant metabolic derangements, and possible aortic rupture, if not treated correctly. These complications likely account for the 43% mortality rate associated with the disorder.^11^ Recently, Pham et al described a new innovative technique in management of aortoduodenal syndrome utilizing endoscopic ultrasound (EUS) guided gastrojejunostomy with lumen-apposing metallic stents (LAMS)^12^ which serve to create a fistula between the stomach and the jejunum to relieve the symptoms of gastric outlet obstruction.^12^

## Conclusion

Aortoduodenal syndrome is a rare clinical entity. Clinicians and radiologists should know that this is a separate entity from the more common SMA syndrome and should know how to differentiate these different pathologies with respect to demographics, clinical history, and imaging findings. Management of these syndromes is also different. Aortoduodenal syndrome should be suspected when patients greater than 60 years of age present with symptoms of gastric outlet obstruction are chronic smokers and/or have a known AAA.

## Learning points

- Learn the difference between superior mesenteric artery (SMA) syndrome and aortoduodenal syndrome.
- High degree of suspicion is required for such rare entity especially when the patient is a chronic smoker and presents with symptoms of gastric outlet obstruction.
- Management options range from conservative therapy to surgical decompression with a promising role of lumen-apposing metallic stents (LAMS).
